# Erianin induces apoptosis and pyroptosis via MAPK/ERK and PI3K/Akt pathways and synergizes with anlotinib in anaplastic thyroid carcinoma

**DOI:** 10.3389/fphar.2025.1596873

**Published:** 2025-09-04

**Authors:** Qiaonan Zhang, Minghan Qiu, Jinpu Liu, Zhanhua Gao, Yuya Liu, Yayun Wang, Ruxue Liu, Zhen Yang, Jie Hao, Ming Gao

**Affiliations:** ^1^ Department of Thyroid and Breast Surgery, Tianjin Union Medical Center, The First Affiliated Hospital of Nankai University, Tianjin, China; ^2^ School of Medicine, Nankai University, Tianjin, China; ^3^ Tianjin Cancer Institute of Integrative Traditional Chinese and Western Medicine, Tianjin Union Medical Center, The First Affiliated Hospital of Nankai University, Tianjin, China; ^4^ Department of Oncology, Tianjin Union Medical Center, The First Affiliated Hospital of Nankai University, Tianjin, China; ^5^ College of Integrative Medicine, Tianjin University of Traditional Chinese Medicine, Tianjin, China

**Keywords:** anaplastic thyroid carcinoma, erianin, caspase-3/GSDME, pyroptosis, anlotinib

## Abstract

**Introduction:**

Anaplastic thyroid carcinoma (ATC) is an aggressive thyroid malignancy characterized by rapid progression, resistance to conventional therapies, and poor patient prognosis. There is an urgent need for innovative therapeutic strategies. Erianin, a natural compound derived from Dendrobium, has demonstrated significant anti-tumor effects in various cancers, yet its role in ATC remains unexplored.

**Methods:**

The anti-tumor effects of erianin were assessed *in vitro* through assays including CCK-8, colony formation, flow cytometry, LDH release, and Western blot. RNA sequencing was conducted for gene expression analysis. Immunofluorescence and transmission electron microscopy evaluated pyroptosis markers. *In vivo* efficacy was validated in CAL62 xenograft mouse models using tumor growth measurement, TUNEL staining, and immunohistochemistry.

**Results:**

Erianin significantly inhibited proliferation and colony formation, induced G2/M cell cycle arrest, apoptosis, and GSDME-dependent pyroptosis in ATC cells. Mechanistically, erianin suppressed activation of the MAPK/ERK and PI3K/AKT signaling pathways. Importantly, erianin synergistically enhanced the anti-tumor efficacy of anlotinib in ATC cells. *In vivo*, combination therapy with erianin and anlotinib resulted in marked tumor growth suppression and increased apoptosis compared to monotherapies.

**Conclusion:**

Collectively, our study demonstrates that erianin exerts potent anti-tumor activity in ATC by simultaneously inhibiting the MAPK/ERK and PI3K/Akt signaling pathways, thereby inducing apoptosis and GSDME-dependent pyroptosis. Furthermore, the synergistic interaction between erianin and anlotinib significantly enhances therapeutic efficacy. These findings position erianin as a promising candidate for the treatment of ATC, offering novel therapeutic insights and supporting further clinical investigations.

## 1 Introduction

Anaplastic thyroid carcinoma (ATC), though rare at approximately 2% of all thyroid cancers, is an aggressive and challenging malignancy with a median overall survival of 4–6 months (Wang J. et al., 2023). Conventional treatments, including chemotherapy, surgery, and radiotherapy, have shown limited success (Wang J. et al., 2023). Genetic studies reveal that ATC is primarily driven by aberrant activation of two critical signaling pathways: the mitogen-activated protein kinase (MAPK) pathway (RAF/MEK/ERK) and the PI3K/Akt pathway (Landa et al., 2016; Pozdeyev et al., 2020). The MAPK pathway plays a vital role in cell growth, proliferation, survival and migration. Among the genetic alterations observed in this pathway, the BRAFV600E mutation is the most common in ATC (Pozdeyev et al., 2020; Califano et al., 2024). Similarly, mutations and dysregulation in PI3K/Akt pathway-related genes are frequently associated with the tumor’s onset, progression, invasiveness, and resistance to therapy (Sementino et al., 2024). Given these findings, targeting the MAPK and PI3K/Akt signaling pathways with pathway-specific inhibitors has emerged as a promising therapeutic strategy for ATC.

BRAF-targeted therapies, such as the combination of dabrafenib and trametinib, have demonstrated promising efficacy in ATC patients with BRAF V600 mutations (Califano et al., 2024). However, treatment options remain limited for cases driven by NRAS mutations or non-V600 BRAF mutations. Tyrosine kinase inhibitors (TKIs), including anlotinib, have emerged as potential alternatives, offering improved outcomes for advanced differentiated thyroid carcinoma (Chi et al., 2023; Kuang et al., 2024). While prospective evidences on ATC are scarce due to its rarity, preclinical and clinical evidence suggests that TKIs provide benefits for ATC patients (Kuang et al., 2024; Ruan et al., 2019; Zheng et al., 2023; Song Y. et al., 2024). Despite these advances, treatment outcomes for ATC remain poor, highlighting the ongoing challenges in managing this aggressive cancer. This underscores an urgent need for innovative therapeutic strategies to overcome current limitations and improve patient prognosis.

Natural product-derived small molecules have become a cornerstone in the development of anti-tumor therapies, offering a rich resource for innovative drug discovery (Atanasov et al., 2021). For example, commonly used chemotherapy drugs such as paclitaxel, etoposide, irinotecan, and vinblastine are derived from natural compounds or their derivatives. Erianin is a natural compound derived from the traditional Chinese medicine Dendrobium, with diverse pharmacological effects such as cell cycle arrest, suppression of cell migration and angiogenesis, and promotion of cell death (Yang et al., 2023; Wei et al., 2024). Studies have shown that erianin exerts significant inhibitory effects on various cancer types (Ma et al., 2023). However, there is limited literature on the anti-tumor effects of erianin in endocrine-related malignancies, and its potential use in treating anaplastic thyroid cancer remains unexplored.

Previous research has shown that erianin exerts anti-tumor activity by inhibiting the MAPK/ERK and PI3K/Akt signaling pathways (Ma et al., 2023), suggesting its potential as a therapeutic agent for ATC. In this study, we confirmed the significant anticancer effects of erianin on ATC through both *in vitro* and *in vivo* models, including the induction of apoptosis and GSDME-dependent pyroptosis in ATC cells. Additionally, our findings revealed that erianin synergistically enhanced the therapeutic efficacy of anlotinib in ATC. These results position erianin as a promising candidate for ATC treatment, providing new avenues for addressing this aggressive malignancy.

## 2 Materials and methods

### 2.1 Cells and reagents

The ATC cell lines CAL62, C643, and BHT101 were purchased from the ATCC and preserved in our laboratory. Cells were maintained at 37 °C and 5% CO_2_ in RPMI-1640 medium (Thermo Fisher Scientific, MA, USA) containing 10% fetal bovine serum (ALLBIO, Zhejiang, China). Erianin was obtained from Shanghai Yuanye Biotechnology Co., Ltd. (Shanghai, China), dissolved in DMSO (Beyotime, Beijing, China) and stored at −80 °C. Anlotinib was purchased from Chia Tai Tianqing Pharmaceutical Group Co., Ltd. (Jiangsu, China).

### 2.2 CCK-8 assay for cell proliferation

The stock solution of erianin (500 μM) was diluted in complete medium to obtain working concentrations of 2.5 nM, 5 nM, 10 nM, 25 nM, and 50 nM. BHT101, C643, and CAL62 cells were plated at a density of 500 cells per well in 96-well plates. After 24 h, different concentrations of drug-containing medium and DMSO-containing control medium were added to the respective groups, with no fewer than three replicates per concentration gradient. From Day 1 to Day 5 after drug addition, CCK-8 reagent (Beyotime, China) was added, incubated in the incubator for 1 h, and the OD values were measured at 450 nm using a microplate reader.

### 2.3 Colony formation assay

Cells in the logarithmic growth phase were plated at 1000 cells per well in six-well plates, followed by treatment with erianin at concentrations of 0, 25 nM, and 50 nM after 24 h. After cross-pattern mixing, the plates were incubated at 37 °C in a 5% CO_2_ humidified incubator for 7 days. Colonies were fixed, stained, photographed, and counted, ensuring at least 50 cells per colony.

### 2.4 CCK-8 assay for determining half-maximal inhibitory concentration (IC50)

Drugs were prepared as serial dilution gradients for subsequent use. Cells in the logarithmic growth phase (BHT101, C643, and CAL62) were plated at 3000 cells per well in 96-well plates, followed by the addition of media containing different drug concentrations after 24 h, with triplicates for each concentration. CCK-8 reagent (Beyotime, China) was added at 48 h, 72 h, and 96 h post-treatment, incubated for 1 h, and OD values were recorded at 450 nm using a microplate reader.

### 2.5 Flow cytometric analysis of cell cycle and cell death

Cells were treated with various concentrations of erianin for 48 h, washed with PBS, and fixed in 70% ethanol. A staining solution containing PI and RNase was added, and cells were incubated at room temperature in the dark for 30 min before analysis by flow cytometry. The Annexin V-FITC/PI dual-staining assay was used to evaluate cell death. Treated cells were centrifuged, washed with PBS, stained with Annexin V-FITC and PI, incubated for 15 min in the dark, and analyzed using a flow cytometer. Data analysis was performed with FlowJo v10.6.2 software.

### 2.6 EdU staining assay

BHT101, C643, and CAL62 cells were seeded in 24-well plates and treated with different drug concentrations after attachment. EdU working solution was added, and the cells were incubated at 37 °C for 2 h. After fixation with 4% paraformaldehyde for 15 min and three PBS washes, the cells were permeabilized with 0.3% Triton X-100. Staining was performed according to the instructions of the EdU detection kit (Beyotime, China). Fluorescence was observed and imaged under a fluorescence microscope, and fluorescence intensity was quantified using ImageJ software.

### 2.7 LDH release assay for cytotoxicity evaluation

LDH cytotoxicity was assessed using an LDH assay kit (Beyotime, China). Cells (1000 cells/well) were seeded in 96-well plates, treated with drugs at various concentrations for 48 h (3 replicates per condition), and the supernatant was removed. LDH release reagent was added, incubated at 37 °C for 1 h, centrifuged, and transferred to new plates. LDH detection solution was added, incubated for 30 min at room temperature in the dark, and absorbance was measured at 490 nm (630 nm as reference) using a microplate reader. Cytotoxicity was calculated as: (% cell death) = (treated absorbance - control absorbance)/(maximum absorbance - control absorbance) × 100.

### 2.8 Western blotting

Proteins were extracted from cells and quantified using the BCA assay. Equal protein amounts were denatured at 95 °C for 5 min, separated by SDS-PAGE, and transferred onto PVDF membranes. Membranes were blocked with 5% skim milk, incubated with appropriately diluted primary antibodies at 4 °C overnight, and washed. HRP-conjugated secondary antibodies were applied for 1 h at room temperature. Protein bands were visualized using ECL reagents and documented with a gel imaging system. Detailed information on the antibodies utilized is provided in Supplementary Table S1.

### 2.9 Transmission electron microscopy

Cells treated with erianin were fixed in 2.5% glutaraldehyde for 2 h at 4 °C, washed with PBS (3 × 10 min), and further fixed in 1% osmium tetroxide for 1–2 h. After washing, cells were dehydrated through an ethanol gradient (30%–100%) and replaced twice with 100% acetone. Samples were embedded in epoxy resin, ultrathin-sectioned to 60–80 nm, and collected on copper grids. Sections were stained with uranyl acetate and lead citrate (15 min each) and observed under a transmission electron microscope.

### 2.10 Bioinformatics analysis

Microarray data ATC samples were obtained from the public GEO database, specifically from the GPL570 platform datasets GSE29265, GSE33640, GSE65144, and GSE76039. Raw data were normalized using the rma algorithm, and batch effects across datasets were corrected with the *limma* package. The integrated dataset included 78 normal thyroid tissues (NT), 69 papillary thyroid carcinoma (PTC), 17 poorly differentiated thyroid carcinoma (PDTC), and 52 anaplastic thyroid carcinoma (ATC) samples. Heatmaps were generated using the *pheatmap* package, and boxplots visualizing gene expression differences were created with the *ggplot2* package.

### 2.11 Immunofluorescence

Cells were seeded on coverslips, treated with erianin, and fixed with 4% paraformaldehyde. N-GSDME primary antibody and fluorescence-conjugated secondary antibody were applied, followed by DAPI staining for nuclei. Membrane localization of N-GSDME was observed under a fluorescence microscope (Nikon). Detailed information on antibodies is provided in Supplementary Table S1.

### 2.12 RNA sequencing

Total RNA was extracted from CAL62 cells using the Trizol method for lysis and purification. RNA libraries were constructed and sequenced on the BGISEQ-500 high-throughput sequencing platform. Differentially expressed genes (DEGs) were identified using the *DESeq2* package. Enrichment analysis was conducted with the *clusterProfiler* package, and results were visualized with the *cnetplot* and *ggplot2* packages.

### 2.13 Combination index (CI) analysis

Cells were seeded at 3000 cells per well in 96-well plates. Based on the individual drug IC_50_ values, erianin and anlotinib were combined in fixed IC_50_ ratio gradients (0, 0.25, 0.5, 1, 2, and 4× IC_50_). Three replicates were set for each combination, along with single-drug treatment and drug-free control groups. After 72 h of treatment, CCK-8 solution was added, and cells were incubated at 37 °C in the dark for 1 h. Absorbance at 450 nm was measured. Combination index (CI) values were calculated using CompuSyn or CalcuSyn software, where CI < 0.9 indicates synergism, 0.9 ≤ CI ≤ 1.1 indicates an additive effect, and CI > 1.1 indicates antagonism (Chou, 2006). Visualization was performed using the ggplot2 package.

### 2.14 Xenograft tumor model

4–6 weeks old BALB/c nude mice were randomly divided into control, erianin, anlotinib, and combination treatment groups. Well-cultured CAL62 anaplastic thyroid cancer cells were mixed with Matrigel (Thermo Fisher Scientific, MA, USA) in a 1:1 volume ratio and subcutaneously injected into the right inguinal area of each mouse (1 × 10^6^ cells/mouse). After 7 days, mice with normally growing solid tumors were allocated to groups and treated as follows: control group received saline; erianin group received 3.5 mg/kg erianin via tail vein injection; anlotinib group received 2 mg/kg anlotinib via intraperitoneal injection; combination group received 2.6 mg/kg erianin via tail vein and 1.5 mg/kg anlotinib via intraperitoneal injection, all administered every other day. After 14 days of treatment, mice were sacrificed, tumor sizes were measured and weighed, and tumor tissues were fixed in formalin for paraffin embedding and subsequent experiments.

### 2.15 Hematoxylin and eosin (HE) staining

Paraffin sections were baked at 65 °C for 2 h, deparaffinized with xylene, rehydrated, and stained with hematoxylin and eosin (HE). After dehydration and clearing, sections were mounted with neutral resin and imaged under a microscope.

### 2.16 TUNEL staining

Paraffin sections were deparaffinized in xylene, rehydrated through graded ethanol, and treated with 20 μg/mL proteinase K for 15–30 min. After PBS washing, TUNEL detection solution (5 μL TdT enzyme +45 μL fluorescein label) was added, and sections were incubated at 37 °C in the dark for 60 min. After PBS washing, slides were mounted with antifade solution and observed under a fluorescence microscope (excitation: 450–500 nm, emission: 515–565 nm).

### 2.17 Immunohistochemistry (IHC) staining

Paraffin sections were deparaffinized, rehydrated through graded ethanol, and subjected to antigen retrieval with citrate buffer under high pressure. After PBS washing, endogenous peroxidase activity was blocked with 3% H_2_O_2_ at room temperature, followed by 5% BSA blocking for 30 min. Primary antibodies were applied and incubated overnight at 4 °C. After PBS washing, HRP-conjugated secondary antibodies were added and incubated at 37 °C for 30 min. DAB was used for chromogenic detection, and nuclei were counterstained with hematoxylin. Sections were dehydrated through graded ethanol, cleared with xylene, mounted with neutral resin, and imaged under a microscope. The antibodies are provided in Supplementary Table S1.

### 2.18 Statistical analysis

Statistical analyses were performed using GraphPad Prism 9.0. Group comparisons were conducted with one-way analysis of variance (ANOVA) and t-tests, with statistical significance set at P < 0.05. Significance levels were denoted as *P < 0.05, **P < 0.01, and ***P < 0.001.

## 3 Results

### 3.1 Erianin inhibits proliferation and induces G2/M phase cell cycle arrest in ATC cells

**FIGURE 1:**
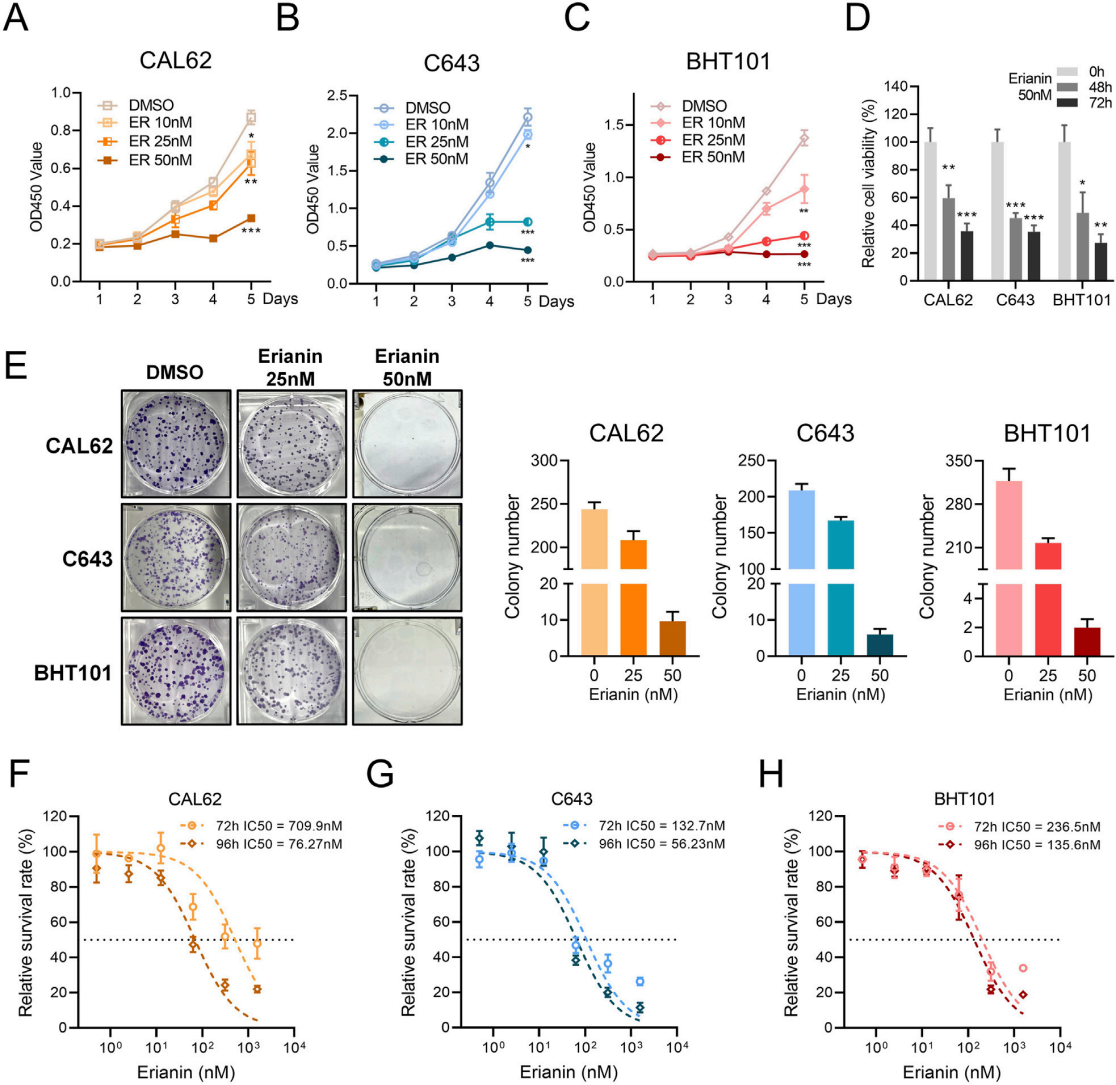
Erianin inhibits proliferation in ATC cells. **(A–C)** The CCK8 assay shows the inhibitory effects of erianin on the proliferation of CAL62 **(A)**, C643 **(B)**, and BHT101 **(C)** thyroid cancer cells, with inhibition observed in a concentration-dependent manner (10 nM, 25 nM, 50 nM) and time-dependent manner. **(D)** The CCK8 assay shows the relative viability of CAL62, C643, and BHT101 cells treated with 50 nM erianin for 48 and 72 h. **(E)** The colony formation assay shows the inhibitory effects of erianin on colony-forming ability in CAL62, C643, and BHT101 ATC cells. **(F–H)** The IC50 values of erianin for CAL62, C643, and BHT101 cells at 72 h and 96 h, as determined by the CCK8 assay. Student’s *t* test was used for testing and analysis; *p < 0.05, **p < 0.01, and ***p < 0.001.

To investigate the suppressive effects of erianin on anaplastic thyroid cancer, we performed *in vitro* assays using ATC cell lines. The CCK-8 assay showed that erianin inhibited the proliferation of CAL62, C643, and BHT101 cells in a concentration- and time-dependent manner (Figures 1A–D). Colony formation assays confirmed this effect, with erianin (25 nM and 50 nM) significantly reducing colony-forming ability in all 3 cell lines in a concentration-dependent manner (Figure 1E). Furthermore, the half-maximal inhibitory concentration (IC50) of erianin was measured for these ATC cell lines. The IC50 values for CAL62, C643, and BHT101 cells at 72 h and 96 h were 709.9 nM, 76.27 nM, 132.7 nM and 56.23 nM, 236.5 nM, 135.6 nM, respectively (Figures 1F–H).

**FIGURE 2:**
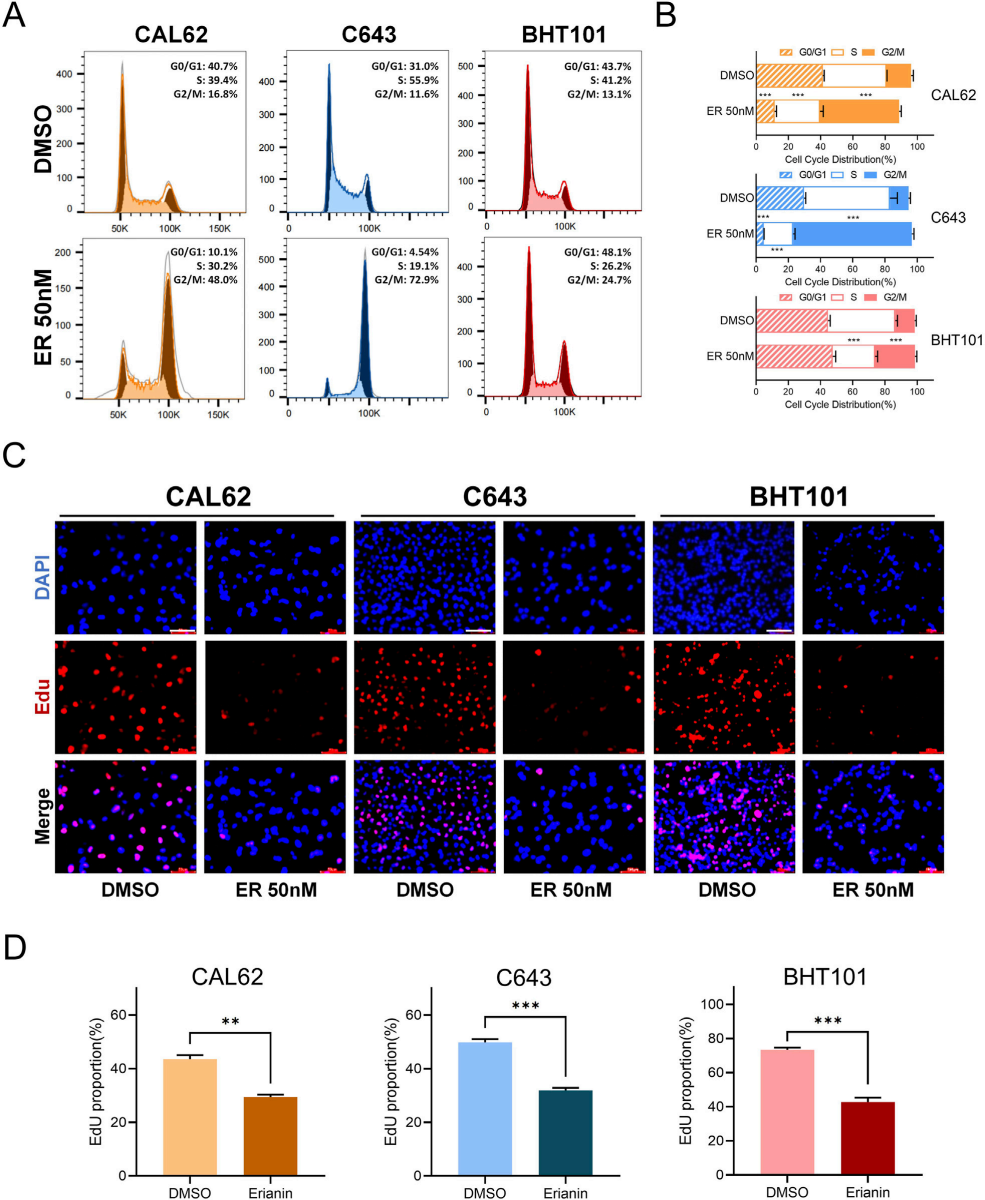
Erianin induces G2/M phase arrest in anaplastic thyroid cancer (ATC) cells. **(A)** Flow cytometry analysis of the cell cycle distribution in CAL62, C643, and BHT101 ATC cells after 48 h of treatment with 50 nM erianin. **(B)** Quantitative analysis of cell cycle distribution in the three ATC cell lines after erianin treatment, presented as bar graphs. **(C)** EdU staining assay shows the inhibitory effect of 50 nM erianin for 48 h on DNA synthesis in the three ATC cell lines. Red fluorescence represents EdU-positive cells, and DAPI staining marks the nuclei. Scale bar: 50 μm. **(D)** Quantitative analysis of EdU-positive rates. Student’s *t* test was used for testing and analysis; *p < 0.05, **p < 0.01, and ***p < 0.001.

We further investigated the effects of erianin on the cell cycle of ATC cells. Flow cytometry revealed significant G2/M phase arrest in CAL62, C643, and BHT101 cells, with increased G2/M phase and decreased G0/G1 and S phase (Figures 2A,B). The EdU assay showed reduced EdU-positive rates in erianin-treated cells, suggesting a strong suppressive effect on DNA synthesis (Figures 2C,D). In conclusion, these results suggest that erianin effectively suppresses the proliferation of anaplastic thyroid cancer cells at nanomolar (nM) levels and induces cell cycle inhibition, leading to G2/M phase arrest, indicating its potential as a therapeutic candidate for thyroid cancer.

### 3.2 Erianin induces apoptosis and GSDME-dependent pyroptosis in ATC cells

**FIGURE 3:**
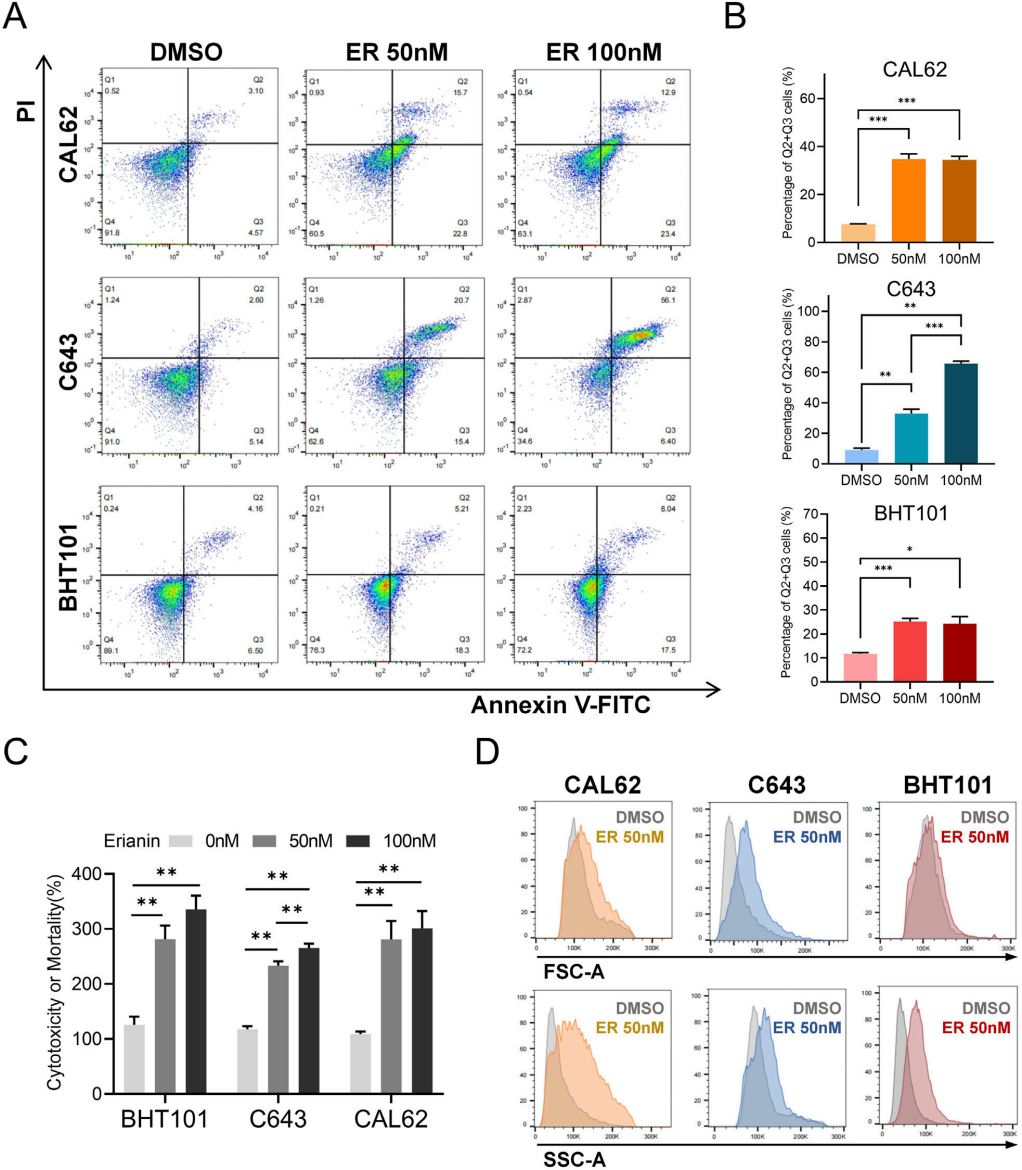
Erianin induces cell death in ATC cells. **(A)** Annexin V-FITC/PI staining shows increased cell death in CAL62, C643, and BHT101 cells treated with 50 nM and 100 nM erianin for 48 h. **(B)** Quantification of cell proportions in the Q2+Q3 quadrants. **(C)** LDH assay confirms the cytotoxic effects of 50 nM and 100 nM erianin on ATC cells after 48 h. **(D)** Flow cytometry reveals changes in cell size (FSC-A) and granularity (SSC-A) in ATC cells treated with 50 nM erianin for 48 h. Student’s *t* test was used for testing and analysis; *p < 0.05, **p < 0.01, and ***p < 0.001.

To evaluate the cytotoxic effects of erianin on ATC cells, Annexin V-FITC/PI staining was used to analyze cell death. Flow cytometry results demonstrated that erianin significantly induced cell death in CAL62, C643, and BHT101 cells. Compared with the DMSO control, treatment with 50 nM and 100 nM erianin significantly increased the proportion of cells in the Q2+Q3 quadrants in a concentration-dependent manner, indicating membrane rupture events (Figures 3A,B). We further measured LDH release in ATC cells following erianin treatment. At 50 nM and 100 nM, erianin markedly enhanced LDH release in CAL62, C643, and BHT101 cells, further corroborating its cytotoxicity (Figure 3C). Interestingly, forward scatter (FSC-A) and side scatter (SSC-A) analysis by flow cytometry indicated increased granularity and cell size in erianin-treated ATC cells (Figure 3D). This observation, inconsistent with the typical apoptotic characteristic of cell shrinkage, indicates the involvement of non-apoptotic mechanisms in erianin-induced cell death.

**FIGURE 4:**
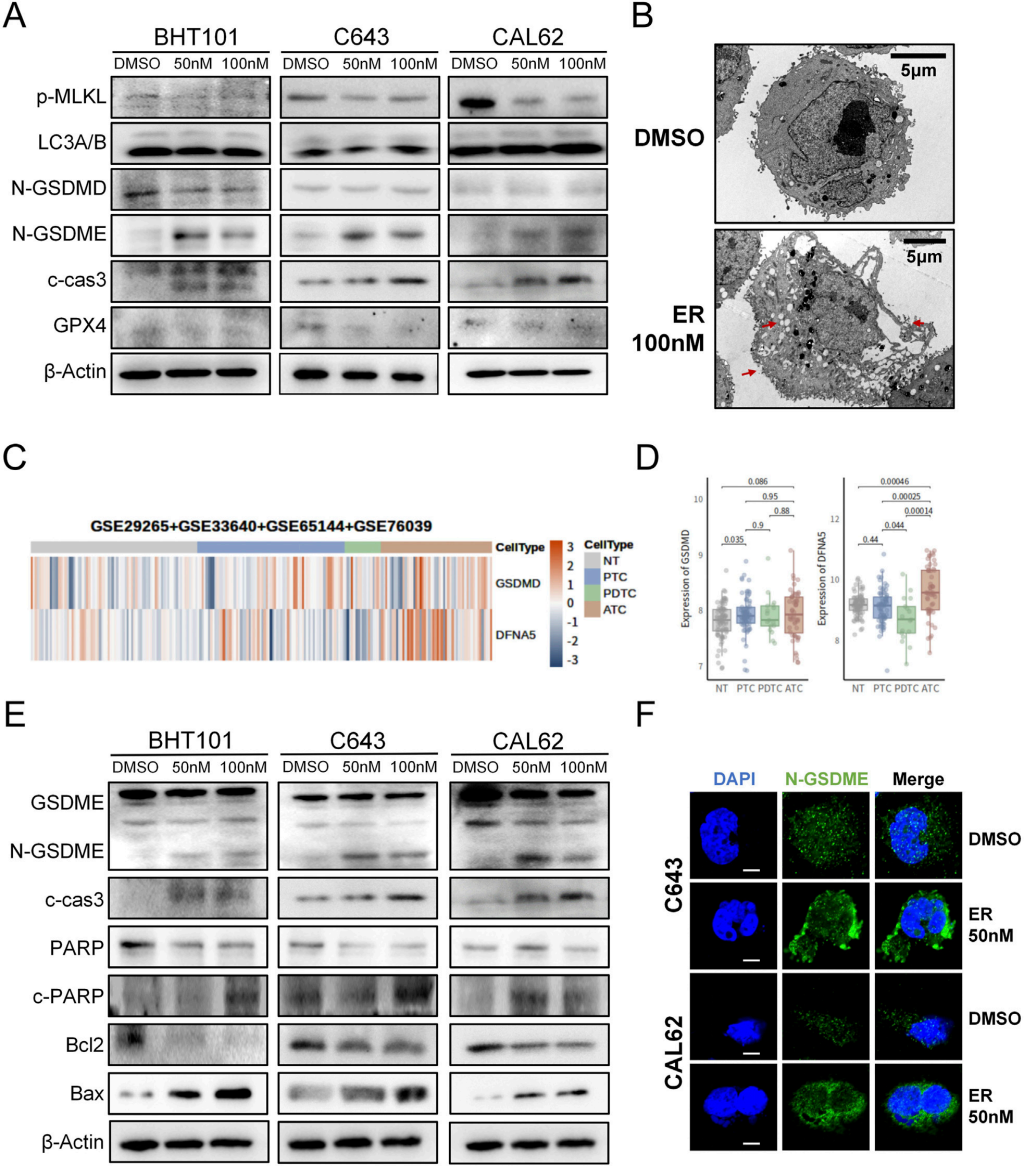
Erianin induces apoptosis and GSDME-dependent pyroptosis in ATC cells. **(A)** Expression levels of key cell death-related proteins in ATC cells following erianin treatment. **(B)** Electron microscopy reveals typical pyroptotic features in erianin-treated cells, including cell swelling, formation of pyroptotic bodies, and membrane pore formation (indicated by arrows). Scale bar: 5.0 μm. **(C)** Heatmap analysis showing the expression levels of GSDME (DFNA5) and GSDMD across different thyroid tissue types. **(D)** Boxplot depicting the expression levels of GSDME (DFNA5) and GSDMD in different thyroid tissue types. **(E)** Expression of caspase-3/GSDME-dependent pyroptosis-related proteins in ATC cells after 48 h of erianin treatment. **(F)** Immunofluorescence analysis of N-GSDME expression and membrane localization in ATC cells after 48 h of erianin treatment. Scale bar: 2.5 μm.

To investigate how erianin induces cell death in ATC cells, we examined the key markers of various cell death pathways. Western blot revealed that erianin significantly increased cleaved caspase-3 and N-GSDME levels (markers of apoptosis and GSDME-dependent pyroptosis), without affecting N-GSDMD (GSDMD-dependent pyroptosis), p-MLKL (necroptosis), LC3A/B (autophagy), or GPX4(ferroptosis) levels (Figure 4A). This suggests erianin induces GSDME-dependent pyroptosis, which characterized by cell swelling and membrane rupture (Liu et al., 2024a). Electron microscopy confirmed pyroptotic morphology, including cell swelling, cytoplasmic vesicle formation, and membrane rupture (Figure 4B), consistent with the cell enlargement observed in flow cytometry. Additionally, by integrating data from ATC-containing samples in the NCBI database, we found that GSDME (DFNA5) was significantly upregulated in ATC samples compared to normal thyroid (NT) and other types of thyroid cancer, whereas GSDMD did not exhibit a consistent upregulation trend (Figures 4C,D). This finding suggests that targeting GSDME-dependent pyroptosis may have greater therapeutic relevance in ATC.

Differing from GSDMD-mediated pyroptosis, GSDME-dependent pyroptosis is predominantly activated via caspase-3 cleavage (Bhat et al., 2023). Western blot further revealed that erianin treatment elevated N-GSDME and cleaved caspase-3 levels, alongside increased the caspase-3 substrate c-PARP expression. Concurrently, Bax was upregulated, while Bcl-2 was significantly downregulated (Figure 4E). These data indicate that erianin regulates the Bcl-2/Bax axis to cleave caspase-3, thereby facilitating GSDME-dependent pyroptosis. GSDME-dependent pyroptosis is initiated by the upregulation of N-GSDME and its membrane localization for pore formation (Liu et al., 2024a). Immunofluorescence analysis demonstrated a marked increase in N-GSDME fluorescence and its aggregation at the plasma membrane in erianin-treated ATC cells compared with controls (Figure 4F). Upon administration of the caspase-3 inhibitor Z-DEVD-FMK, we found that blocking caspase-3 activity partially alleviated erianin-induced cell death. At the protein level, Z-DEVD-FMK was also found to partially inhibit the expression of cleaved caspase-3 and the N-terminal fragment of GSDME (Supplementary Figure S1). Collectively, these results suggest that erianin exerts its cytotoxic effects on ATC cells by inducing apoptosis and GSDME-dependent pyroptosis through the activation of caspase-3.

### 3.3 Erianin suppresses the MAPK/ERK and PI3K/AKT pathways in ATC cells

**FIGURE 5:**
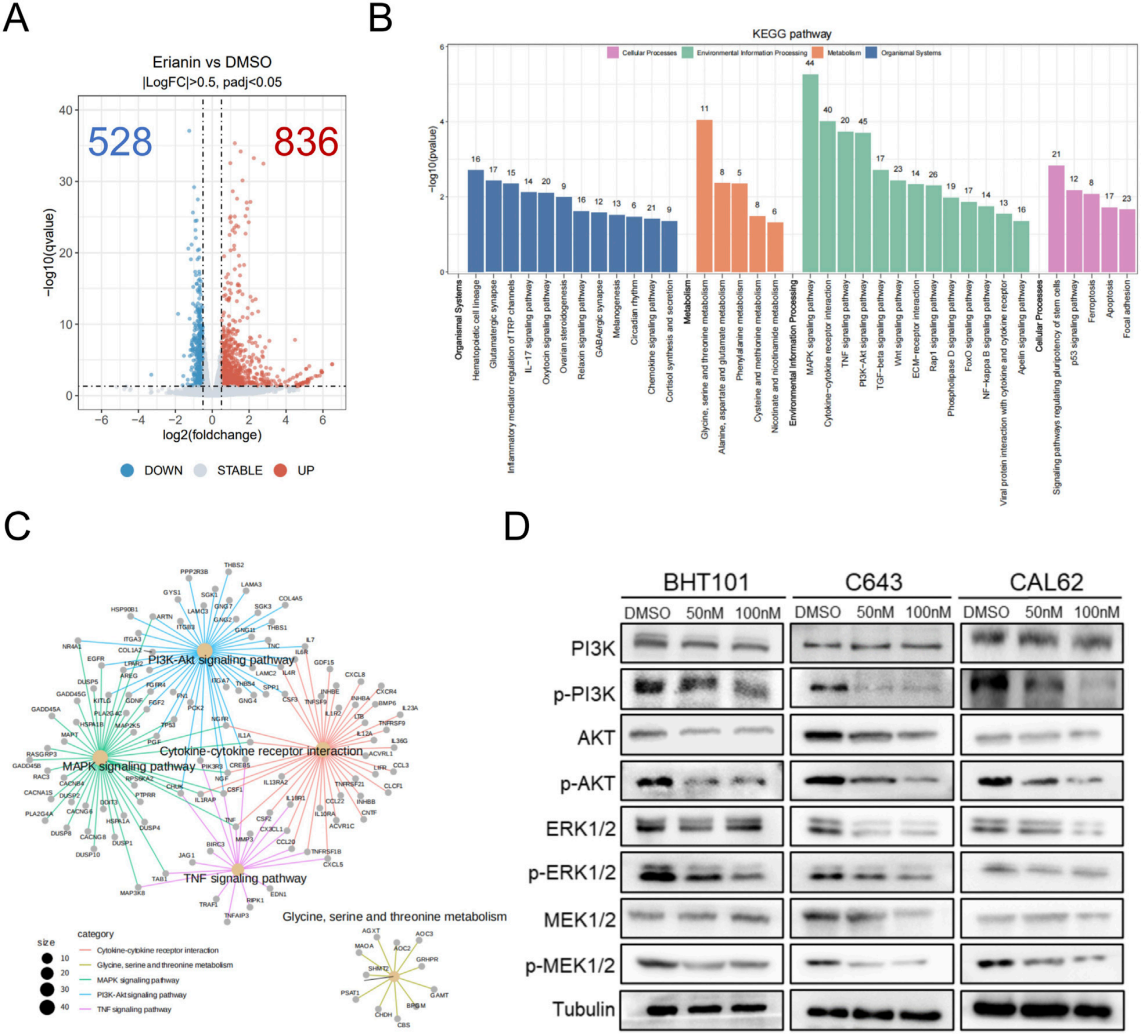
Erianin suppresses the MAPK/ERK and PI3K/AKT pathways in ATC cells. **(A)** Volcano plot of differentially expressed genes (DEGs) in CAL62 cells treated with 50 nM Erianin for 48 h. **(B)** KEGG enrichment analysis of the DEGs. **(C)** Cnet enrichment network of KEGG pathway analysis results. **(D)** Expression of PI3K/AKT and MAPK/ERK pathway-related proteins after 48-h treatment with different concentrations of Erianin (0, 50 nM, 100 nM).

To investigate how erianin inhibits ATC cells, RNA-seq was performed on CAL62 cells treated with 50 nM erianin for 48 h and controls. A total of 1364 DEGs were identified, with 836 upregulated and 528 downregulated (Figure 5A, Supplementary Material 1). KEGG enrichment analysis revealed that Erianin affects tumor-related pathways, particularly the MAPK and PI3K/AKT pathways (Figures 5B,C, Supplementary Material 2). Both the MAPK and PI3K/AKT pathways are classical oncogenic pathways that regulate caspase-3 activity. Therefore, we further validated erianin’s effects on these pathways at the protein level. Western blot analysis showed that erianin significantly reduced the phosphorylation levels of key proteins in the MAPK/ERK pathway (ERK1/2, MEK1/2) and the PI3K/AKT pathway (PI3K, AKT) in all three ATC cell lines. Additionally, the total protein expression levels of these pathway components also showed a downward trend (Figure 5D). These results suggest that Erianin inhibits MAPK/ERK and PI3K/AKT pathway activity, potentially contributing to its suppression of ATC cell malignancy.

### 3.4 Erianin synergistically enhances the efficacy of anlotinib in ATC

Anlotinib is a multi-target receptor tyrosine kinase inhibitor that primarily exerts its antitumor effects by inhibiting pathways such as VEGFR in various cancers (Song et al., 2020). It also exhibits promising antitumor activity in anaplastic thyroid carcinoma (ATC), but its therapeutic efficacy remains limited (Song Y. et al., 2024). Resistance to anlotinib has been associated with aberrant activation of the PI3K/AKT signaling pathway (Chen et al., 2022a), motivating an investigation into whether erianin could potentiate its inhibitory effects.

**FIGURE 6:**
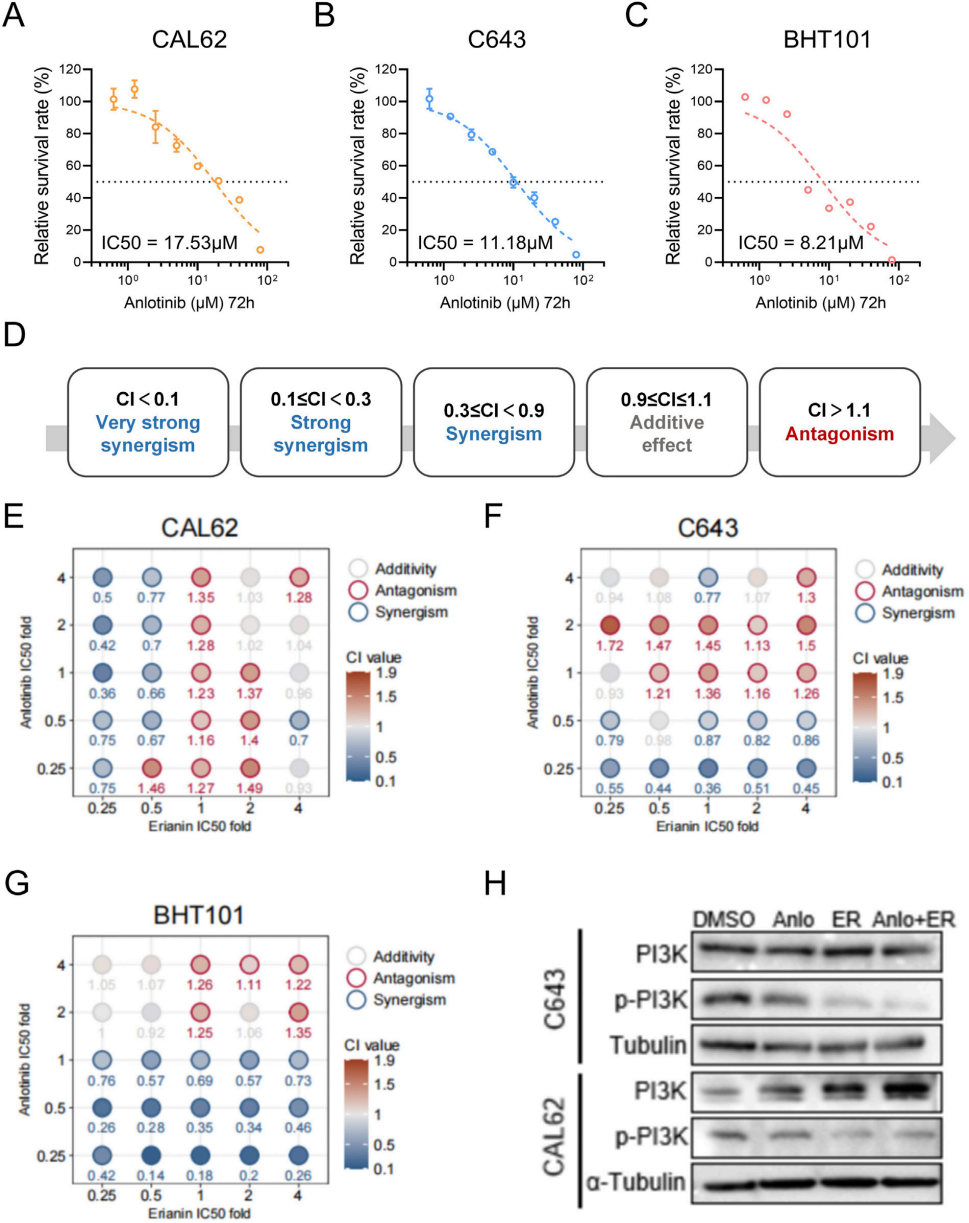
Anlotinib and Erianin exhibit synergistic effects in treating ATC cells. **(A–C)** The 72 h IC50 values of anlotinib for BHT101, C643, and CAL62 cells. **(D)** Classification of Combination Index (CI) values: CI < 0.1 indicates very strong synergy; 0.1 ≤ CI < 0.3, strong synergy; 0.3 ≤ CI < 0.9, synergy; 0.9 ≤ CI ≤ 1.1, additive effect; and CI > 1.1, antagonism. **(E–G)** Heatmaps of CI values for Erianin and anlotinib at fixed ratio gradients (0, 0.25, 0.5, 1, 2, 4× IC50) in CAL62, C643, and BHT101 cells, respectively. CI < 0.9 (blue) indicates synergy, while CI > 1.1 (red) indicates antagonism. **(H)** Western blot showing p-PI3K protein levels in ATC cells under treatments of DMSO (control), 50 nM Erianin (ER), 2 μM anlotinib (Anlo), or the combination (50 nM ER + 2 μM Anlo).

Using CCK-8 assays, the 72-h IC50 of anlotinib was determined as 17.53 μM for CAL62, 11.18 μM for C643, and 8.21 μM for BHT101 cells (Figures 6A–C), while erianin’s IC50 values were 236.5 nM, 132.7 nM, and 727.3 nM, respectively (Figures 1F–H). To further evaluate the synergistic effects of the Erianin and anlotinib, we conducted Combination Index (CI) analysis (Chou, 2006) using fixed ratio gradients (0, 0.25, 0.5, 1, 2, 4× IC50) of the two drugs (Figure 6D). The results showed significant synergistic effects (CI < 0.9) between erianin and anlotinib at multiple concentrations, particularly at low concentrations (0.25× IC50), in all three ATC cell lines (Figure 6E). Western blot analysis demonstrated that the combination treatment markedly enhanced the suppression of p-PI3K compared to single-agent treatments (Figure 6H). Collectively, these findings indicate that erianin significantly potentiates the anti-tumor efficacy of anlotinib in ATC cells *in vitro*, achieving notable synergy even at low doses, thereby highlighting its potential clinical applicability.

### 3.5 Erianin suppresses ATC tumor growth and enhances the therapeutic efficacy of anlotinib *in vivo*

**FIGURE 7:**
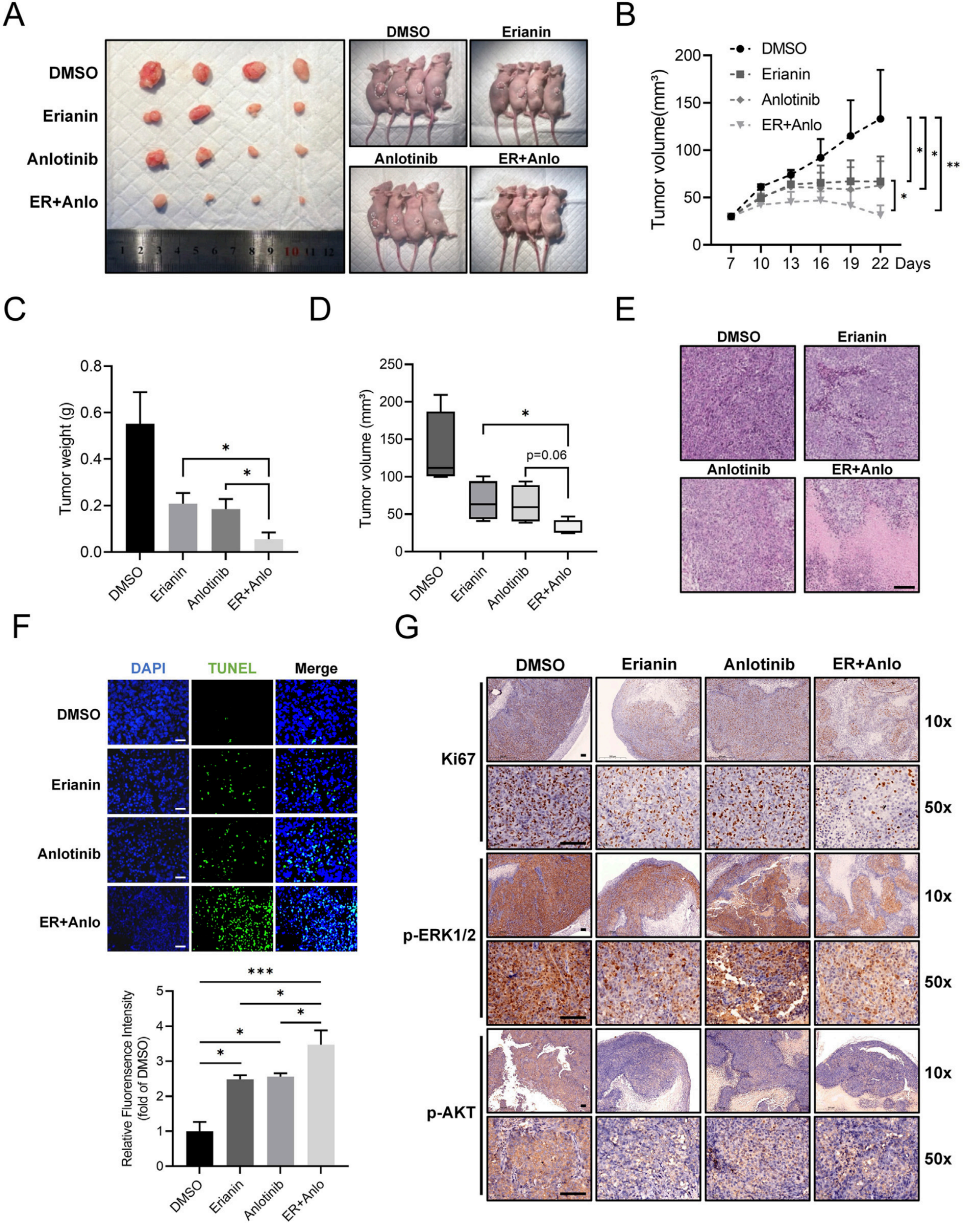
Erianin suppresses ATC tumor growth and enhances the therapeutic efficacy of anlotinib *in vivo*. **(A)** Representative images of tumors and tumor-bearing nude mice from DMSO, erianin, anlotinib, and combination treatment groups. **(B)** Growth curves of tumor volumes in mice under different treatments. **(C,D)** Quantification of tumor weights and final tumor volumes across treatment groups. **(E)** HE staining analysis of tumor tissues. Scale bar: 200 μm. **(F)** TUNEL staining of tumor tissues. Scale bar: 100 μm. **(G)** Immunohistochemistry for Ki67, p-ERK1/2, and p-AKT in tumor tissues. Scale bar: 100 μm.

To validate the *in vitro* findings, a subcutaneous CAL62 ATC xenograft model in nude mice was used to assess the *in vivo* effects of erianin. Tumor growth was significantly slower in both erianin and anlotinib monotherapy groups compared to controls, with the combination therapy showing the strongest inhibition (Figures 7A–D). HE and TUNEL staining revealed increased tumor cell death and apoptosis in both monotherapy groups, with the combination therapy group showing the most pronounced effects (Figures 7E,F). IHC confirmed the drugs' inhibitory effects on tumor proliferation and signaling pathways (Figure 7G). Both monotherapies reduced Ki67-positive cells and p-ERK1/2 and p-AKT levels, with the combination therapy causing further reductions. Collectively, these findings further validate the inhibitory effects of Erianin on ATC *in vivo* and reveal its significant synergistic anti-tumor effects with anlotinib.

## 4 Discussions

Anaplastic thyroid cancer (ATC) is a rare but highly aggressive thyroid malignancy, accounting for 1.3%–9.8% of all thyroid cancers but contributing to 30%–50% of thyroid cancer-related deaths (Wang J. et al., 2023). The median survival of ATC is only 5 months, with a 1-year survival rate of approximately 20%, and very few patients surviving beyond 2 years after diagnosis (Wang J. et al., 2023; Califano et al., 2024). Due to its rapid progression and poor response to conventional treatments such as surgery, radiotherapy, and chemotherapy, ATC remains a significant clinical challenge.

Traditional Chinese medicine contains numerous bioactive small molecules with significant anticancer properties. Dendrobium is a classic medicinal herb that has been used in China for over 1,500 years (Zhang et al., 2023). Erianin, the main active ingredient in Dendrobium, has attracted significant attention due to its potent anti-tumor effects in various malignancies (Yang et al., 2023). Studies have shown that erianin possesses anti-tumor, immunomodulatory, anti-inflammatory, antibacterial, and antiviral activities (Yang et al., 2023; Wei et al., 2024; Ma et al., 2023). Regarding its anticancer properties, Erianin has been shown to inhibit the progression of multiple cancers, such as colorectal cancer, lung cancer, breast cancer, liver cancer, melanoma etc. (Ma et al., 2023; Wang P. et al., 2023; Chen et al., 2020; Zheng et al., 2024). Currently, research on erianin’s effects in endocrine tumors is limited. Our team previously showed that erianin inhibits pancreatic cancer progression via AKT and ASK1 targeting (Liu R. et al., 2024). However, no studies have yet investigated the effects of Erianin on ATC.

In this study, we demonstrate for the first time that Erianin significantly inhibits ATC cell proliferation both *in vitro* and *in vivo*, induces cell cycle arrest, and triggers apoptosis and GSDME-dependent pyroptosis. Our study demonstrates that erianin exerts strong cytotoxicity against ATC cell lines, with IC50 values in the nanomolar range. As shown in our previous studies (Liu R. et al., 2024) and other reports (Yang et al., 2023; Wang P. et al., 2023; Chen et al., 2020), erianin consistently shows nanomolar-level activity across various tumors. Compared with currently used targeted agents for ATC, such as dabrafenib, trametinib, and anlotinib—which generally show IC50 values in the micromolar range—erianin demonstrates markedly enhanced efficacy (Birden et al., 2022). Its IC50 values are more comparable to those of classic chemotherapeutic agents like gemcitabine, paclitaxel, and doxorubicin (Chung et al., 2011; Voigt et al., 2005). Although this study remains in the preclinical stage and direct comparisons with standard clinical therapies may be premature, the low IC50 values of erianin highlight its potential as a promising therapeutic candidate for ATC. Further research is warranted to improve its solubility and tumor-targeting capacity.

Pyroptosis is a novel form of programmed cell death characterized by cell swelling, the formation of dense intracellular vacuoles, membrane perforation, and eventual rupture (Liu et al., 2024a). GSDM family proteins are essential regulators of pyroptosis, as their cleaved N-terminal fragments form pores in the plasma membrane, leading to cell lysis (De Schutter et al., 2021). Among the GSDM family, GSDMD and GSDME are the most extensively studied members, with distinct activation mechanisms: GSDMD is activated via inflammasome-mediated cleavage, whereas GSDME is directly cleaved and activated by caspase-3 (Lawlor et al., 2024). Pyroptosis mediated by the caspase-3/GSDME axis indicates that certain apoptosis-inducing anticancer drugs may also possess the potential to induce pyroptosis (Liu et al., 2024c). Analysis of public datasets revealed that GSDME expression is elevated in ATC tissues compared to normal thyroid tissue and other thyroid cancer subtypes. Although further experimental validation is required, this observation suggests that GSDME-dependent pyroptosis may play a critical role in enhancing drug sensitivity in ATC, as previously noted in the literature (Zhao et al., 2022). Notably, we found that erianin significantly induces caspase-3/GSDME-dependent pyroptosis in ATC cells. Unlike other thyroid cancer subtypes with relatively low malignancy, ATC is highly aggressive and exhibits pronounced anti-apoptotic features (Molinaro et al., 2017). As an alternative cell death pathway, erianin-induced GSDME-dependent pyroptosis may provide a novel strategy to overcome therapeutic resistance in ATC.

MAPK and PI3K/AKT are the most frequently implicated pathways driving ATC progression (Landa et al., 2016). In ATC, aberrant activation of the MAPK/ERK signaling pathway is highly prevalent, and combinations of BRAF and MEK inhibitors targeting this pathway have demonstrated significant efficacy and safety in ATC treatment (S et al., 2024). Additionally, sustained activation of the PI3K/AKT pathway is a critical mechanism promoting ATC cell proliferation and apoptosis resistance (S et al., 2024; Xu and Ghossein, 2016). Studies have shown that MAPK/ERK pathway activation directly inhibits caspase-3 activity, while PI3K/AKT activation modulates apoptosis-related proteins through multiple pathways, ultimately affecting cell death (Wang et al., 2020). In our study, we found that erianin significantly attenuated the malignant biological behaviors of ATC cells by simultaneously inhibiting the MAPK/ERK and PI3K/AKT pathways. This dual-pathway synergy not only effectively suppressed ATC cell proliferation *in vitro* but also reinforced erianin’s antitumor effects by enhancing cell death pathways.

Anlotinib is a multi-target tyrosine kinase inhibitor which has shown promising efficacy in the treatment of ATC (Ruan et al., 2019; Zheng et al., 2023; Song Y. et al., 2024; Liang et al., 2021). A clinical trial evaluating anlotinib-based regimens for locally advanced or metastatic ATC reported an objective response rate exceeding 60%, with most adverse events being well-tolerated (Zheng et al., 2023). However, the therapeutic efficacy of anlotinib monotherapy is limited, and resistance commonly develops, significantly restricting its clinical utility. This resistance has been reported to be often associated with aberrant activation of the PI3K/AKT signaling pathway (Chen et al., 2022a; Chen et al., 2022b). In this investigation, Combination Index (CI) analysis demonstrated a significant synergistic interaction between erianin and anlotinib, with enhanced inhibition of the PI3K/AKT pathway. These findings suggest that erianin complements the limitations of anlotinib monotherapy, significantly improving overall antitumor efficacy. Notably, this synergy occurs at lower doses of both agents, highlighting their potential for dose optimization in combination therapy.

Although this study demonstrated the antitumor effects of erianin and its combination with anlotinib against ATC through both *in vitro* and *in vivo* analyses, several limitations remain. First, the specific targets through which erianin inhibits the MAPK/ERK and PI3K/AKT pathways have not been fully elucidated. Second, a comprehensive evaluation of the toxicity and pharmacokinetics of erianin and its combination therapy was not conducted. Future studies should focus on *in vivo* pharmacokinetic assessments and long-term toxicity analyses to determine the safe dosage range and potential side effects of erianin, supporting its clinical translation. Finally, this study observed that erianin induces GSDME-dependent pyroptosis in ATC tumor cells, suggesting it may enhance antitumor immunity by releasing immunostimulatory molecules. As a combination agent, anlotinib also possesses the ability to modulate the tumor immune microenvironment, and their synergistic interaction may further enhance the efficacy of immunotherapy (Song F. et al., 2024). Future investigations could explore its potential to improve ATC immunotherapy outcomes when combined with immune checkpoint inhibitors.

Overall, this study is the first to elucidate the role and mechanisms of erianin in ATC: erianin inhibits the MAPK/ERK and PI3K/AKT signaling pathways, inducing caspase-3-dependent apoptosis and GSDME-dependent pyroptosis, and demonstrates synergistic potential when combined with anlotinib. These findings provide a novel therapeutic option for ATC and offer foundational insights for developing more effective treatment strategies.

## Data Availability

The dataset supporting the conclusions of this study is available at the NCBI GEO repository (https://www.ncbi.nlm.nih.gov/geo/query/acc.cgi?acc=GSE299493).
