# Supplementary material for: Erianin induces apoptosis and pyroptosis via MAPK/ERK and PI3K/Akt pathways and synergizes with anlotinib in anaplastic thyroid carcinoma
Source: Front Pharmacol. 2025 Sep 4;16:1596873. doi: 10.3389/fphar.2025.1596873 (PMC12443827; doi:10.3389/fphar.2025.1596873)
Supplement: Supplementary file 2 [file Supplementaryfile3.docx]

**Supplementary Figure and Tables**

**Erianin induces apoptosis and pyroptosis via MAPK/ERK and PI3K/Akt pathways and synergizes with anlotinib in anaplastic thyroid carcinoma**

Qiaonan Zhang^1, 4, #^, Minghan Qiu ^2, 3, #, *^, Jinpu Liu ^1, 4, #^, Cheng Feng^1, #^, Zhanhua Gao^1, 4^, Yuya Liu^1, 4^, Yayun Wang^5^, Ruxue Liu^5^, Xiangqian Zheng^6^, Zhen Yang^2^, Jie Hao^1, 2, *^, Ming Gao^1, 2, *^

1. Department of Thyroid and Breast Surgery, Tianjin Union Medical Center, The First Affiliated Hospital of Nankai University, Tianjin 300121, China.
2. Tianjin Cancer Institute of Integrative Traditional Chinese and Western Medicine, Tianjin Union Medical Center, The First Affiliated Hospital of Nankai University, Tianjin 300121, China.
3. Department of Oncology, Tianjin Union Medical Center, The First Affiliated Hospital of Nankai University, Tianjin 300121, China.
4. School of Medicine, Nankai University, Tianjin, 300071, China.
5. College of Integrative Medicine, Tianjin University of Traditional Chinese Medicine, Tianjin 300121, China.
6. Department of Thyroid and Neck Tumor, Tianjin Medical University Cancer Institute and Hospital, National Clinical Research Center for Cancer, Key Laboratory of Cancer Prevention and Therapy, Tianjin's Clinical Research Center for Cancer, Tianjin, 300060, China.

#Qiaonan Zhang, Minghan Qiu, Jinpu Liu and Cheng Feng contributed equally to this work.

Correspondence to:

Ming Gao, headandneck2008@126.com; Jie Hao, haojie1215@126.com; Minghan Qiu, qiuminghan@163.com.

**Supplementary Table 1**

| Antibody | experiment | company | Catalog no. |
| --- | --- | --- | --- |
| Bax | Western blot | Abclonal Technology | A19684 |
| Bcl-2 | Western blot | Abclonal Technology | A19693 |
| cleaved caspase-3 | Western blot, IF | Cell Signaling Technology | 9664 |
| AKT | Western blot | Cell Signaling Technology | 9272 |
| p-AKT | Western blot, IHC | Cell Signaling Technology | 4060 |
| ERK1/2 | Western blot | Cell Signaling Technology | 9102 |
| p-ERK1/2 | Western blot, IHC | Cell Signaling Technology | 4377 |
| GPX4 | Western blot | Proteintech Group | 30388-1-AP |
| Ki67 | IHC | Affinity Biosciences | AF0198 |
| LC3A/B | Western blot | Cell Signaling Technology | 4108 |
| MEK1/2 | Western blot | Proteintech Group | 11049-1-AP |
| p-MEK1/2 | Western blot | Cell Signaling Technology | 9154 |
| N-GSDMD | Western blot | abcam | ab215203 |
| GSDME | Western blot, IF | abcam | ab215191 |
| N-GSDME | Western blot, IF | abcam | ab222408 |
| PARP | Western blot | Cell Signaling Technology | 9532 |
| cleaved PARP | Western blot | Cell Signaling Technology | 5625 |
| PI3K | Western blot | Proteintech Group | 60225-1-Ig |
| p-PI3K | Western blot | Cell Signaling Technology | 4228 |
| p-MLKL | Western blot | Cell Signaling Technology | 18640 |
| Tubulin | Western blot | Proteintech Group | 66009-1-Ig |
| β-actin | Western blot | Proteintech Group | 66031-1-Ig |


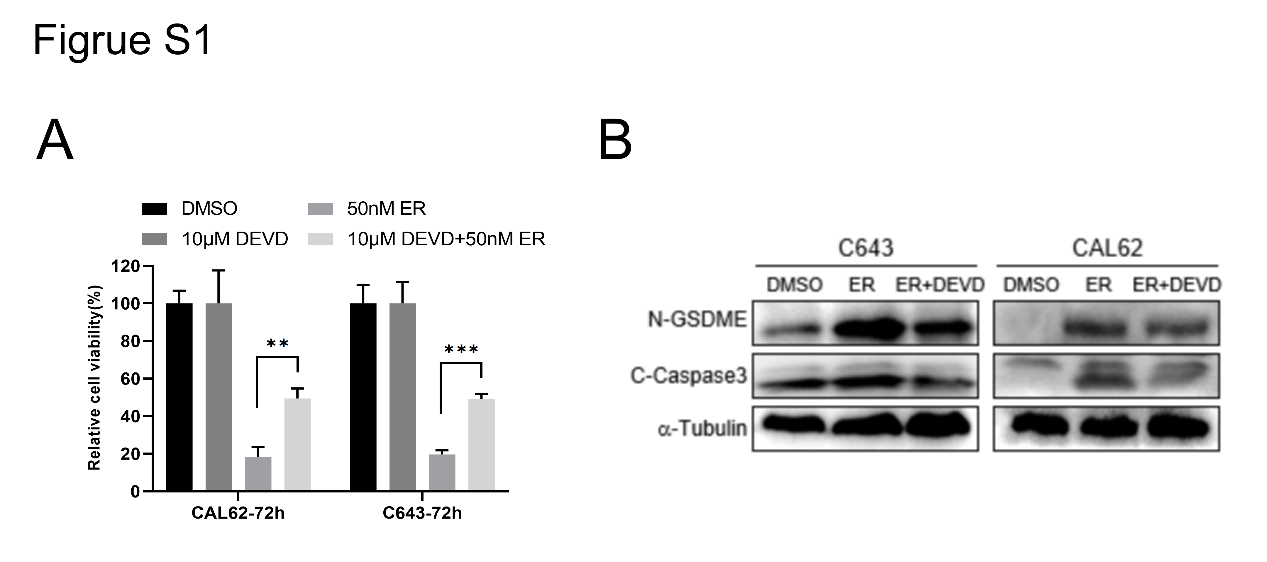


**Figure S1. Inhibition of caspase-3 partially reverses erianin-induced cell death and suppresses GSDME cleavage. (A)** Cell viability of CAL62 and C643 anaplastic thyroid carcinoma (ATC) cells treated with DMSO, 50 nM erianin (ER), 10 μM Z-DEVD-FMK (DEVD), or a combination of 50 nM ER and 10 μM DEVD for 72 hours, as assessed by CCK-8 assay. **P < 0.01, *P < 0.001; two-tailed Student’s t-test. (**B)** Western blot analysis of N-GSDME and cleaved caspase-3 in C643 and CAL62 cells treated as in (A). Z-DEVD-FMK partially suppressed ER-induced cleavage of caspase-3 and GSDME.
